# A study on the climate-driven spatiotemporal dynamics of influenza in Lanzhou spanning the COVID-19 era

**DOI:** 10.3389/fcimb.2026.1765305

**Published:** 2026-02-23

**Authors:** Hong Shi, Na Zhang, Huan Wei, Haojun Liang, Hui Zhang, Huimin Zhang, Biao Wang

**Affiliations:** 1 Microbiology Laboratory, Lanzhou Center for Disease Control and Prevention, Lanzhou, China; 2 Gansu Provincial People’s Hospital, Lanzhou, China; 3 Gansu Provincial Center for Disease Control and Prevention, Lanzhou, China; 4 Public Health School, Gansu University of Chinese Medicine, Lanzhou, China

**Keywords:** COVID-19 pandemic, influenza, Lanzhou China, meteorological factors, spatiotemporal evolution

## Abstract

**Objective:**

The COVID-19 pandemic has profoundly altered global influenza circulation. This study aimed to examine how meteorological factors influenced influenza transmission in Lanzhou, China, across three distinct phases: before, during, and after the COVID-19 pandemic.

**Methods:**

We collected weekly influenza surveillance data and corresponding meteorological indicators for Lanzhou from January 2014 to December 2024. An explainable machine-learning framework integrating XGBoost with Shapley Additive exPlanation (SHAP) values was used to quantify the dynamic impact of environmental factors on influenza virus positivity rates.

**Results:**

From 2014 to 2019, influenza circulation in Lanzhou followed typical northern hemisphere seasonality, with annual winter–spring peaks usually dominated by a single subtype. From 2020 to 2024, however, influenza activity displayed a clear “disruption-to-reconstruction” trajectory. During the COVID-19 pandemic (2020–2022), stringent non-pharmaceutical interventions (NPIs) caused influenza positivity rates and case numbers to collapse, with seasonal peaks nearly disappearing. In the post-pandemic period (2023–2024), influenza epidemics reemerged, but the environmental drivers of transmission—particularly for the dominant Influenza A/H3N2 subtype—shifted substantially. SHAP analyses and relative-contribution assessments consistently showed that environmental influences were strongly masked by NPIs during the pandemic, resulting in markedly reduced explanatory power. After NPIs were lifted, preliminary observation environmental effects resurfaced but in a reshaped pattern: temperature became the predominant driver, with its contribution increasing to nearly 40%, while the influence of humidity, sunshine, and other factors weakened. Although the characteristic winter peak persisted before and after the pandemic, the previously complex, multifactorial environmental model simplified into a more temperature-centric structure in the post-pandemic era.

**Conclusion:**

This study demonstrates that COVID-19 not only temporarily interrupted influenza transmission but also altered the long-term ecological drivers of influenza. Post-pandemic influenza epidemics are entering a new phase, now dominated by a simplified temperature-centered environmental model, suggesting that the climate–influenza relationship has changed after probably major societal intervention. Thus, in Lanzhou and similar climates, the effectiveness of early warning systems based on historical static models requires reassessment and dynamic recalibration. Future influenza surveillance and forecasting will require more flexible frameworks that integrate multi-source data—environmental factors, viral evolution, population immunity, and social behavior—to better address the evolving infectious disease ecosystems.

## 1 Introduction

Influenza is an acute respiratory disease caused by influenza viruses (Nypaver et al., 2021), which are characterized by high mutation rates and rapid transmission. Owing to the widespread susceptibility to influenza infection, outbreaks and epidemics can easily occur (Forleo-Neto et al., 2003). Influenza poses a significant global public health threat, affecting approximately 10% of adults and 20% of children each year. This results in approximately 1 billion cases, 3–5 million hospitalizations, and 290,000–650,000 respiratory deaths (Iuliano et al., 2018; Qi et al., 2020; Paget et al., 2023). Seasonal influenza imposes a substantial public health burden in China, contributing to an average of 88,000 additional respiratory deaths annually (Fu et al., 2024). Climatic factors, including temperature, humidity, and atmospheric pressure, affect the stability and transmission rates of respiratory viruses. They may also affect host immune responses, including innate and adaptive immune reactions to respiratory infections, contributing to heightened viral activity during autumn and winter (Moriyama et al., 2020). The COVID-19 pandemic coincided with the peak season for respiratory viruses, such as influenza (Leuzinger et al., 2020). To combat the global COVID-19 pandemic, governments implemented NPIs, including lockdowns, travel restrictions, social distancing, mandatory mask-wearing, and school and shopping mall closures to curb SARS-CoV-2 transmission. While these measures effectively controlled SARS-CoV-2 spread, they also significantly altered the transmission dynamics of other respiratory viruses, notably influenza (Park et al., 2021; Leuzinger et al., 2021; Tempia et al., 2021; Chow et al., 2023). Significant changes in seasonal influenza transmission patterns have been observed in Northwest China (Wang et al., 2025). Related studies have indicated that post-pandemic influenza virus detection rates show an overall upward trend compared to pre-pandemic levels, suggesting potential alterations in viral ecology and transmission dynamics (World Health Organization, 2023). This phenomenon warrants further investigation to understand its underlying drivers and the potential implications of these epidemiological shifts for public health. Due to China’s vast territory, complex climatic conditions, and large population, seasonal variations in influenza activity are pronounced across different regions (Li et al., 2019). Previous studies have indicated that influenza epidemics in temperate regions exhibit distinct seasonal patterns (Du et al., 2022).

Lanzhou, a typical mountain-basin city in northwest China, is characterized by closed valley topography with low wind speeds and frequent temperature inversions. The region has a dry, temperate, continental climate with scarce precipitation and high evaporation. These conditions favor the accumulation of air pollutants and respiratory droplets in the basin and may prolong the survival and transmission of respiratory viruses, such as influenza. Therefore, Lanzhou and the surrounding northwest region provide a suitable setting for investigating how local climatic and topographical factors shape influenza dynamics. Since the global outbreak of COVID-19 in late 2019, nations have implemented NPIs to curb SARS-CoV-2 transmission, altering environmental exposure and host contact patterns (Huang et al., 2021). These interventions may have reshaped the relationship between environmental factors and influenza infection risk, necessitating a reassessment of our understanding of these dynamic associations in the post-pandemic era.

This study aimed to elucidate the complex interactions between environmental factors and influenza virus transmission dynamics, focusing on three distinct phases in Lanzhou City, Northwest China: pre-pandemic, pandemic, and post-pandemic. Through phase-based analysis, we sought to capture the temporal evolution of the influence of climate factors on influenza transmission, particularly under the significant disruption caused by NPIs. Employing advanced machine learning techniques, including XGBoost models and the Shapley additive interpretation framework (Zhu et al., 2022), we quantified the contributions of environmental drivers, such as temperature, humidity, wind speed, sunshine duration, atmospheric pressure, precipitation, and mean temperature difference, to influenza positivity rates. This methodology enables us to distinguish the independent and interactive effects of climate variability and pandemic interventions on influenza subtype activity, filling critical gaps in existing knowledge. This study aims to enhance adaptive responses to evolving environmental changes and public health risks while improving future influenza epidemic forecasting, early warning, and integrated prevention and control capabilities.

## 2 Materials and methods

### 2.1 Influenza surveillance data

The data for this study were obtained from the Chinese Influenza Surveillance Information System, specifically the influenza surveillance data for Lanzhou from January 1, 2014, to December 31, 2024. This dataset includes daily influenza virus test volumes, detection rates, and predominant circulating subtypes (Influenza viruses are classified according to the WHO naming conventions: influenza A(H1N1)pdm09 and A(H3N2), and influenza B Victoria lineage. For brevity, these viruses are hereinafter referred to as H1N1, H3N2, and B/Victoria, respectively) in Lanzhou City. The positivity rate for each influenza subtype was calculated using the following formula:


$$\text{Positive Rate subtype(\%)}(\%)=\frac{\text{ Npositive},\text{ subtype}}{/\text{Ntested}}\times 100\%$$

To analyze the impact of meteorological factors on the transmission of influenza subtypes, we divided the study period into three distinct phases based on the timeline of the COVID-19 pandemic in Lanzhou, China.

- Pre-pandemic Period (January 1, 2014, to December 31, 2019): This refers to the period before SARS-CoV-2 was detected in Lanzhou, China, during which no non-pharmaceutical interventions were implemented.
- Pandemic Period (January 1, 2020, to December 31, 2022): Public health interventions were implemented during this phase.
- Post-Pandemic Period (January 1, 2023, to December 31, 2024): Zero-COVID policies were relaxed during this phase.

### 2.2 Meteorological data

Meteorological data are publicly accessible after registration on the official website of the National Meteorological Science Data Center (data.cma.cn/). The meteorological factors included the weekly average temperature (°C), weekly average temperature difference (°C), weekly average air pressure (hPa), weekly average precipitation (mm), weekly average relative humidity (%), weekly average sunshine duration (h), and weekly average wind speed (m/s).

### 2.3 Data preprocessing

To systematically evaluate the epidemiological patterns of influenza viruses from 2014 to 2024, particularly the impact of NPIs, we conducted multidimensional statistical analyses encompassing descriptive, seasonal visualization, nonlinear, and interactive approaches. The study period was divided into three phases for intergroup comparisons: pre-COVID (2014–2019), during the COVID-19 pandemic (2020–2022), and post-COVID era (2023–2024). Both influenza surveillance and meteorological data were integrated into weekly time series for subsequent analysis. Weekly weather data were matched to the positivity rates of each influenza subtype. Before modeling, all time series underwent missing data checks. Isolated or consecutive missing values ≤2 weeks were imputed using linear interpolation to ensure consistency across datasets.

### 2.4 Data analysis

Data processing and statistical analyses were performed using the R4.5.3 software. Normality was assessed using the Shapiro-Wilk test, and homogeneity of variance was evaluated using Levene’s test. When the data were normally distributed with equal variances, ANOVA was applied; when normally distributed but with unequal variances, Welch’s ANOVA was used. The Kruskal-Wallis test was used for continuous variables that did not meet the normality assumption. *Post-hoc* pairwise comparisons were performed using the Wilcoxon signed-rank test for normally distributed data and the Mann-Whitney U test for non-normally distributed data. All pairwise comparisons were performed using the Bonferroni correction for multiple comparisons. Categorical variables were compared between groups using the chi-square or Fisher’s exact test. Statistical significance was set at P < 0.05. Figures were generated using GraphPad Prism software (version 8.0).

### 2.5 Explainable machine learning analysis

To quantify the independent contribution of meteorological factors to influenza positivity rates and analyze their spatiotemporal heterogeneity, this study employed an interpretable machine learning framework based on XGBoost and SHAP. The XGBoost algorithm excels at handling structured data and time series, demonstrating particular strengths in managing missing data and capturing complex nonlinear interactions among predictor variables (Zhang et al., 2025). Prediction modeling with XGBoost: The model employs cross-validation and early stopping to select the optimal iteration round, reporting R² and RMSE for regression scenarios. SHAP values for each observation were computed using the TreeExplainer algorithm of XGBoost, which assigns marginal contributions to model predictions based on the Shapley value theory from game theory (Wang et al., 2021). SHAP values are interpreted as follows: SHAP value > 0 indicates that the feature increases the predicted influenza positivity rate; SHAP value < 0 indicates that the feature decreases the predicted positivity rate; the absolute magnitude of the SHAP value reflects the strength of the feature’s contribution. To assess the impact of the COVID-19 pandemic on the meteorological-influenza association, independent XGBoost-SHAP models were constructed for three periods: Pre-COVID-19 (2014–2019), COVID-19 (2020–2022), and Post-COVID-19 (2023–2024). The relative importance and directional changes in meteorological factors across these periods were compared. The SHAP summary plot visualizes the global feature contribution distribution for each meteorological factor, where the point color indicates the feature value magnitude (red = high, blue = low), and the x-axis represents the SHAP values (impact direction and strength).

## 3 Results

### 3.1 XGBoost model performance

Our XGBoost model demonstrated relatively robust explanatory power during the early stages of the pandemic, but limited explanatory power during the pandemic and post-pandemic periods. The R² value exceeded 0.15 across all three periods, with the RMSE fluctuating between 0.1350 and 0.1600. Details are provided in **Supplementary Table S1**. Given this, we adopted a conservative approach in interpreting the model’s SHAP values, primarily using them to identify the relative importance and direction of influence of variables, rather than treating the absolute contribution rates reported within the model as direct evidence of the magnitude of causal effects.

### 3.2 Epidemiological dynamics of seasonal influenza in Lanzhou

**Figure 1:**
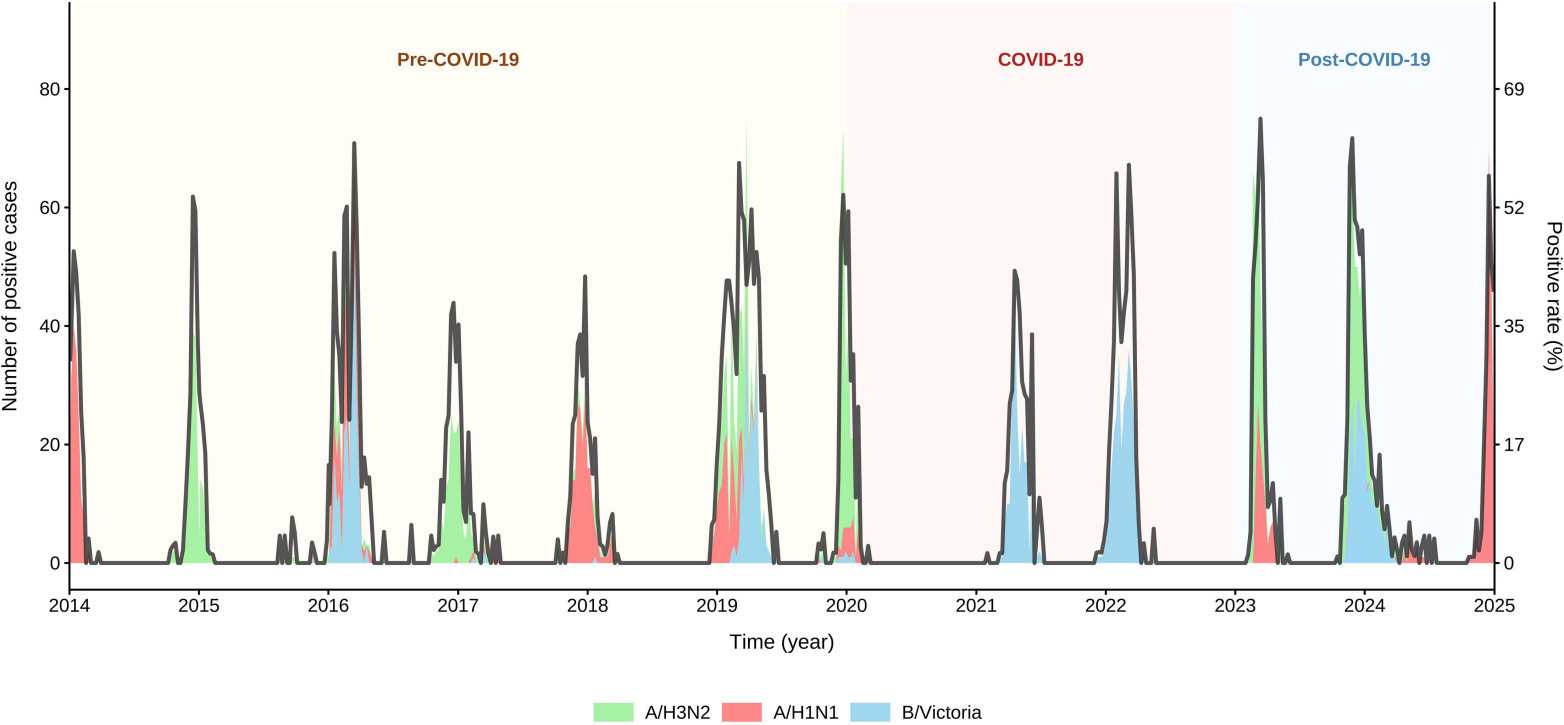
Temporal trends in the number of cases and positive rates for the three influenza A subtypes and B lineage (A/H3N2, A/H1N1,B/Victoria) from 2014 to 2024.

Before the COVID-19 outbreak, influenza virus circulation in Lanzhou, China, exhibited typical seasonal patterns of the northern hemisphere, with annual peaks occurring from winter to the following spring. These outbreaks are usually dominated by a single subtype, despite the coexistence of multiple subtypes. The data revealed distinct antigenic drift patterns among the subtypes. A/H3N2 dominated in most years, whereas B/Victoria exhibited a relatively stable circulation intensity. Notably, A/H1N1 exhibited intermittent outbreak characteristics. However, during 2020-2021, all subtypes experienced epidemiological silence, consistent with the suppression of influenza transmission by non-pharmaceutical interventions against COVID-19. Subsequently, only the B/Victoria subtype was circulating during the 2021–2022 season. In the post-pandemic era, as national control measures were lifted and epidemic management gradually normalized, the 2023–2024 influenza season exhibited a typical, high-intensity seasonal peak occurring during the winter months from late 2023 to early 2024. The peak magnitude of positive cases indicated a significant rebound in overall influenza activity levels, returning to the pre-COVID-19 pandemic typical intensity and breaking free from the abnormal suppression observed between 2020 and 2022. The A/H3N2 subtype once again became the predominant circulating subtype, constituting the largest proportion and accounting for the vast majority of positive cases during the season. The influenza A(H1N1)pdm09 and influenza B Victoria subtypes were also detected, but their activity levels remained relatively low, maintaining a co-circulating status. As shown in **Figure 1**.

**Figure 2:**
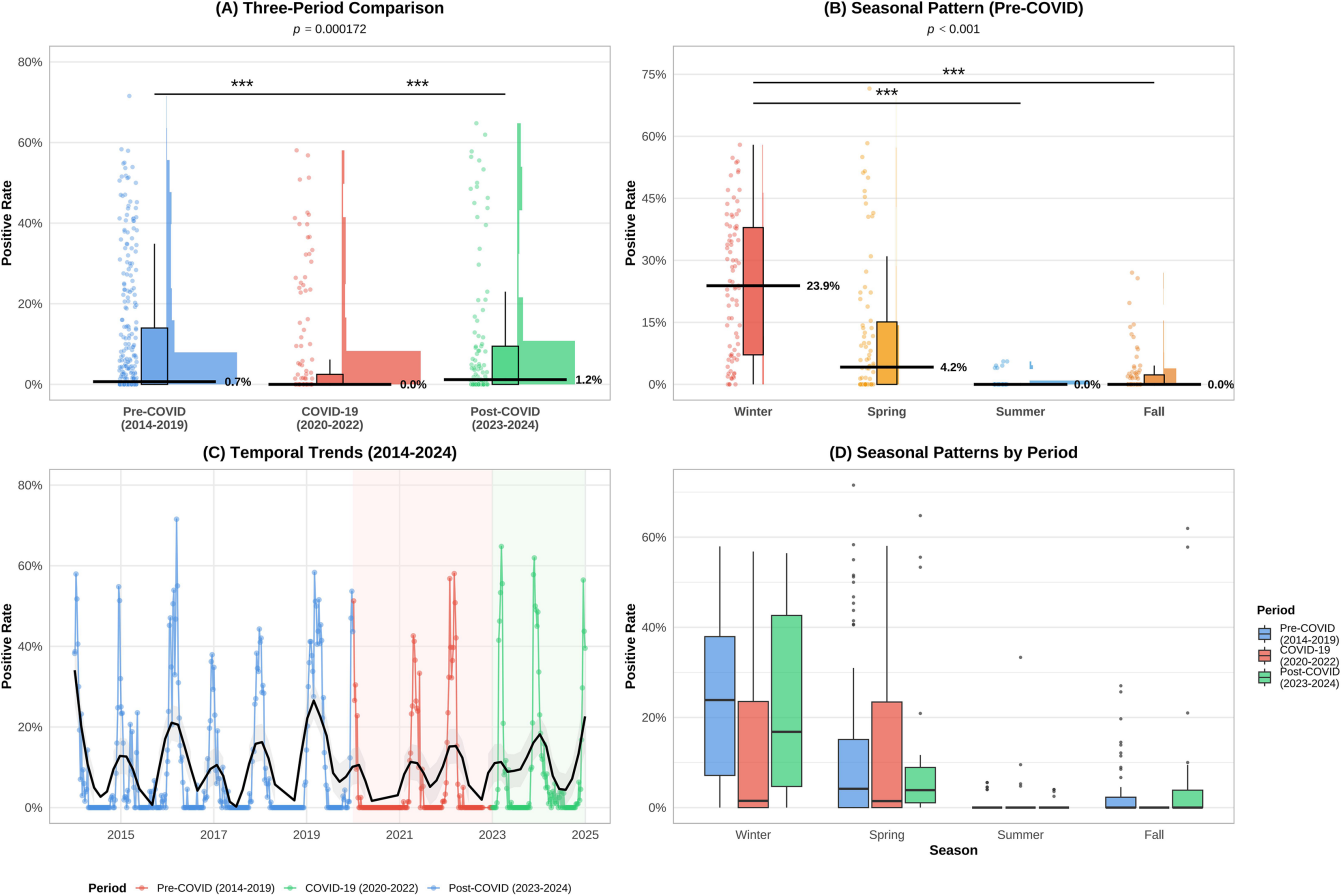
Spatiotemporal patterns of influenza positive rates from 2014 to 2024, incorporating **(A)** three-period comparisons, **(B)** pre-COVID-19 seasonal variation, **(C)** long-term temporal trends across pandemic phases(blue: Pre-COVID-19; red: COVID-19; green: Post-COVID-19); black line indicates smoothed overall trend. **(D)** period-stratified seasonal patterns with statistical significance.* Indicates statistical significance (p<0:05), *** Indicates statistical significance (p<0:001),n.s. Indicates statistical significance.

The results indicated that during the pre-COVID period, influenza positivity rates remained elevated, with a median of approximately 15%, reflecting typical seasonal influenza transmission patterns. During the COVID-19 period, the influenza positivity rate significantly declined to approximately 5%, indicating that NPIs exerted a strong suppressing effect on influenza transmission. In the post-COVID period, the influenza positivity rate rebounded to approximately 10%, as shown in **Figure 2A**. This suggests a gradual resumption of influenza virus transmission. Statistical analysis revealed highly significant differences between the periods (p < 0.001), as shown in **Supplementary Table S2**. Further analysis of the pre-pandemic data revealed seasonal patterns. The positivity rate exhibited a classic winter peak, with the median winter positivity rate (23.9%) significantly higher than that in spring (4.2%), summer, and autumn (0%) (p = 0.000172), confirming the strong seasonality of influenza. As shown in **Figure 2B**. The time-series trend clearly revealed regular winter peaks from 2014 to 2019 (**Figure 2C**), a sharp decline and near disappearance of epidemic intensity from 2020 to 2022, and the re-emergence of epidemic peaks in 2023–2024. The smoothing curve overlaid on the line graph highlights this overall “disruption-recovery” trend. To further investigate whether seasonal patterns changed across different periods, we plotted the phased seasonal boxplots. As shown in **Figure 2D**. The results indicate that interphase differences in winter positivity rates were highly statistically significant (p < 0.001), while seasonal patterns in spring and summer showed no significant changes across phases (p > 0.05), as detailed in **Supplementary Table S3**. This suggests that NPIs primarily influence the intensity of influenza activity during the traditional winter season rather than its fundamental transmission patterns throughout the year.

### 3.3 Impact of environmental factors on influenza incidence

**Figure 3:**
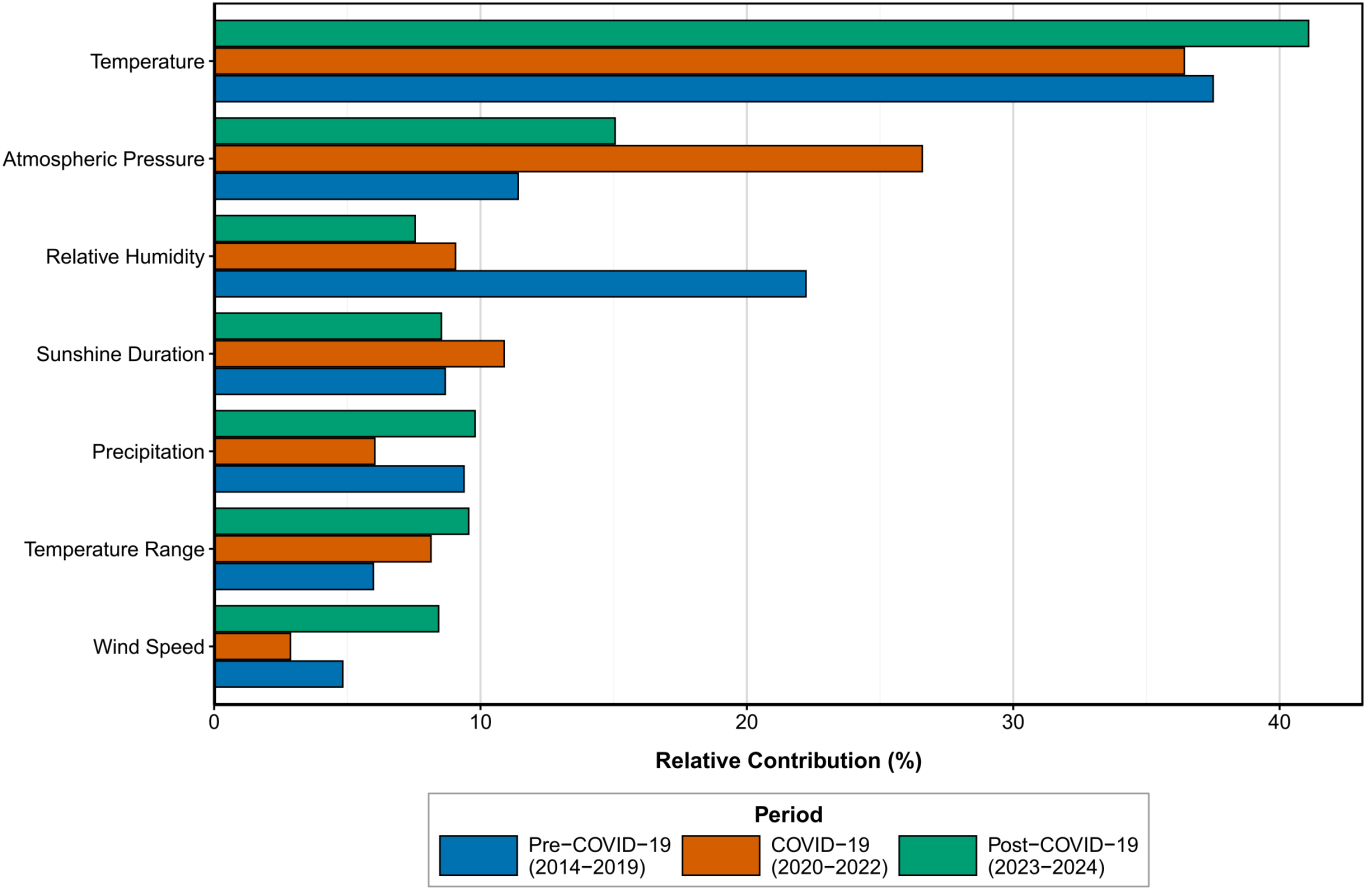
Relative contribution of environmental factors to influenza comparative analysis across COVID−19 pandemic periods.

Before, during, and after the COVID-19 pandemic, we observed significant differences in the contributions of environmental factors across the different phases of the outbreak, with changes in multiple factors being particularly pronounced. To illustrate this, we constructed grouped-level bar charts showing the relative percentage contributions of each factor during the pre-pandemic, pandemic, and post-pandemic periods. As shown in **Figure 3**, the findings reveal a shift from a multi-factor-driven pattern in the pre-pandemic phase to overall suppression during the pandemic phase, followed by selective reconstruction in the post-pandemic phase. The succession and stability of the dominant factors suggest that temperature tended to remain one of the key contributors across all three periods. Its contribution was comparatively high in the pre-pandemic phase, while its relative contribution rate increased to nearly 40% in the post-pandemic phase, indicating a more prominent role among the environmental variables.Atmospheric pressure exhibited robust secondary dominance across periods, indicating a stable underlying association with influenza transmission that remains largely unaffected by intense social interventions. Among the other meteorological factors, relative humidity, sunshine duration, and temperature difference demonstrated moderate explanatory power in the pre-pandemic phase. However, their contributions significantly diminished during the pandemic, reflecting the marginalization of natural environmental drivers under high-intensity social interventions. This relative contribution analysis strongly demonstrates that the COVID-19 pandemic, as a global natural experiment, profoundly reshaped the strength of the epidemiological associations between environmental factors and influenza transmission (**Supplementary Table S4**).

**Figure 4:**
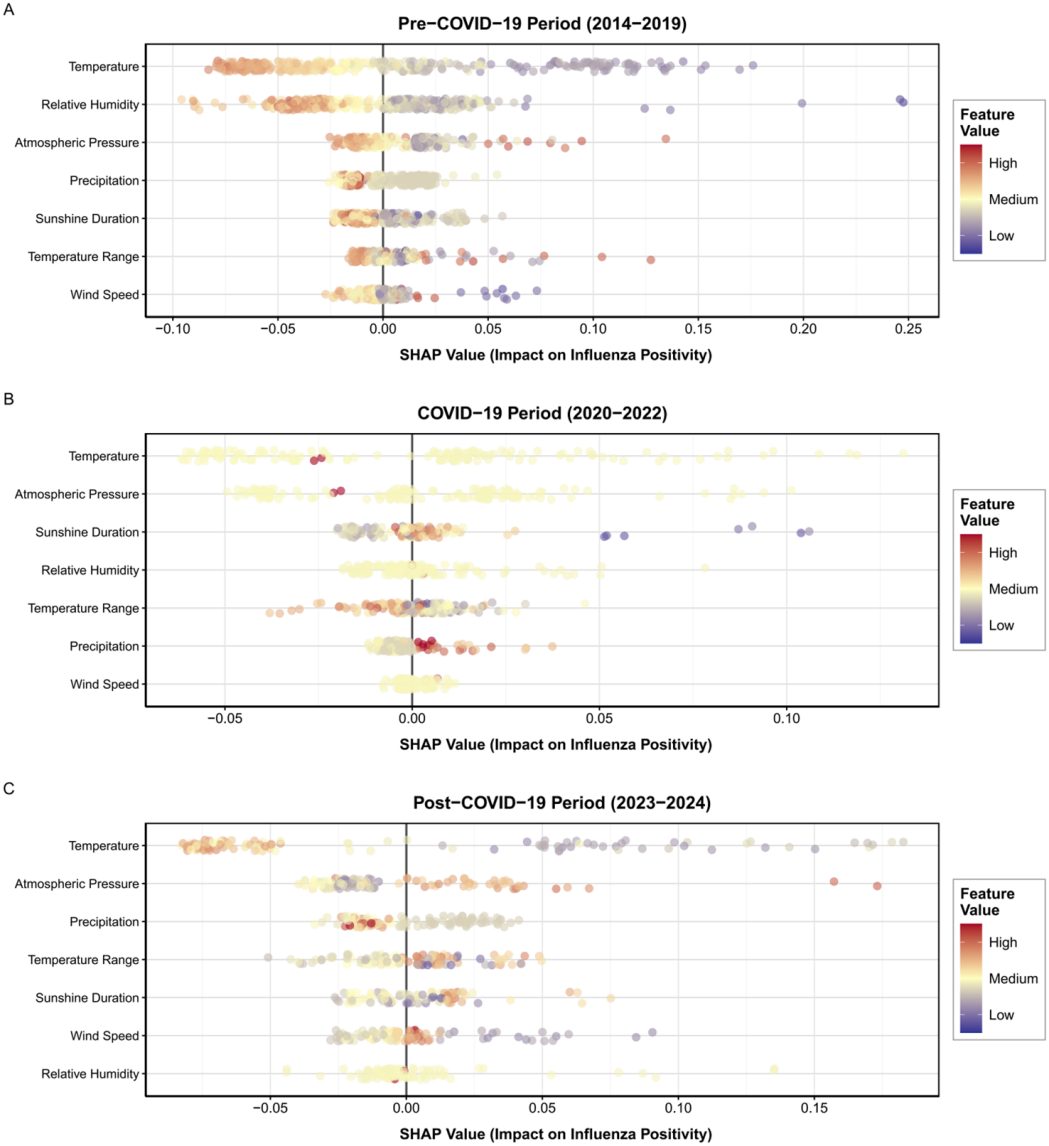
SHAP summary of environmental impacts on influenza positivity across periods: **(A)** 2014–2019 (Pre-COVID-19), **(B)** 2020–2022 (During COVID-19), **(C)** 2023–2024 (Post-COVID-19). Y-axis: meteorological features; X-axis: SHAP values (feature contribution); point color = feature value (high=red, low=blue).

To comprehensively reveal the dynamic changes in environmental factors influencing influenza transmission, we employed an XGBoost model combined with SHAP analysis. The results indicate that the pre-epidemic period was dominated by environmental drivers, exhibiting a classic environmental-driven pattern. The wide distribution range of the SHAP values across features suggests the strong explanatory power of environmental factors for influenza transmission. Temperature was the most influential predictor. Its point cloud distribution was the broadest, with SHAP values ranging from approximately -0.05 to 0.22, revealing a clear dose-response relationship. Low temperatures (blue points) were densely clustered in regions with high positive SHAP values, indicating that cold temperatures were a primary driver of influenza epidemics. Conversely, high temperatures (red points) predominantly corresponded to negative SHAP values, indicating an inhibitory effect. Sunshine Duration exhibited a synergistic effect with temperature. Shorter sunshine duration (blue points) correlated with an increased positive risk, consistent with shorter daylight hours in winter and increased indoor activity. The effects of Relative Humidity and Atmospheric Pressure are relatively complex, with scattered point cloud distributions indicating potentially nonlinear relationships with positivity rates or modulation by other factors. Precipitation, Temperature Range, and Wind Speed exhibited the weakest influence during this period, with SHAP values clustered around zero. See **Figure 4A**.

The COVID-19 pandemic was dominated by social interventions. The most notable feature of this phase was the dramatic narrowing of the distribution range of the SHAP values for all environmental factors, with values highly clustered around zero. Although the ranking of each feature of influence remained unchanged, its absolute impact significantly diminished. Taking the most important feature, “temperature,” as an example, its SHAP value range contracted from approximately 0.27 units to less than 0.1 units, as shown in **Figures 4A, B**. The color distribution pattern in the point cloud shifted from a blue-to-red gradient associated with temperature and became blurred, indicating that the previously strong correlation between environmental factors and the positivity rate was severely weakened. This pattern provides compelling evidence that under robust non-pharmaceutical interventions (e.g., mask mandates, social distancing, and travel restrictions), social behavioral factors completely overshadow the natural driving effects of environmental factors on influenza transmission. Consequently, the predictive role of meteorological conditions was insignificant, as shown in **Figure 4B**.

Preliminary observations in the post-pandemic period indicate that we are currently in a transitional and restructuring phase of the study. During this stage, the influence of environmental factors shows localized recovery, but their patterns have undergone significant evolution. The impact of temperature has rebounded, with its SHAP value distribution range widening considerably compared to the pandemic period (see **Figures 4B, C**), although it has not yet fully recovered to pre-pandemic levels (as shown in **Figure 4A**). This suggests that traditional seasonal factors are returning, although their intensity may be weakened by persistent behavioral changes. The relative ranking of the influence of atmospheric pressure has significantly increased, making it the second most important factor after temperature. Its point cloud distribution has broadened, with high pressure (red dots) correlating with positive SHAP values. This may reveal a novel meteorological-disease linkage mechanism under the “new normal” that warrants further investigation. Other factors, such as precipitation and wind speed, showed limited recovery in influence, with their SHAP value distributions remaining relatively concentrated (**Figure 4C**).

## 4 Discussion

This study systematically analyzed the dynamic evolution patterns of influenza virus positivity rates in Lanzhou, China, during the pre-pandemic, pandemic, and post-pandemic periods. By integrating the XGBoost model with SHAP analysis, we precisely quantified the relative impact of meteorological factors on transmission dynamics. The findings revealed significant differences in the influenza virus circulation patterns and environmental sensitivities across distinct pandemic phases. Our findings reveal a distinct “disruption-reconstruction” process in influenza activity, which is consistent with the results of Zhao et al. (2025). During the early epidemic phase, influenza circulation exhibited strict seasonal patterns, alternating between A/H3N2 and A/H1N1 strains, consistent with the dominant seasonal influenza patterns observed in southern China (Zhang et al., 2025). This aligns with the classic mechanism by which viruses maintain circulation through antigenic drift under the pressure of herd immunity. However, robust NPIs during the pandemic induced an “epidemiological silence” in influenza transmission (Gascó-Laborda et al., 2024), with influenza virus positivity rates dropping to low levels, and the circulation of all subtypes being nearly completely suppressed. This “disruption” demonstrated the effectiveness of NPIs in curbing influenza transmission while simultaneously disrupting the long-term ecological niche structure of influenza viruses in the short term (Chen et al., 2024). The findings of this study must be interpreted within the broader context of evolving virological and population immunological dynamics. Notably, during the 2023–2024 influenza season, dominated by the A/H3N2 subtype, post-pandemic circulation did not revert to pre-pandemic baseline levels, an observation consistent with corresponding reports from Switzerland (Gosert et al., 2025). Despite A/H3N2 re-emerging as the dominant strain and exhibiting a typical winter peak, we observed a significant shift in the pattern of environmental drivers for the first time. Based on SHAP analysis and relative contribution assessments, the relative impact of temperature in the post-pandemic era was abnormally amplified, accounting for nearly 40% of the contribution and becoming the dominant environmental factor. This suggests that the composition of seasonal drivers for influenza may have undergone structural reorganization or simplification during the post-pandemic phase. Concurrently, the model’s low R² value indicates that beyond meteorological factors, other confounding variables—such as non-pharmaceutical interventions, shifts in population behavior, adjustments to testing strategies, and viral evolution itself—may exert greater influence on influenza variability in the post-pandemic era. Therefore, although certain meteorological variables ranked highly in the SHAP ranking within this study, this result should be interpreted as reflecting consistency in the relative importance and directional associations among variables rather than as an absolute explanation for epidemic intensity or proof of causal effects. We have explicitly outlined the interpretive limitations of SHAP analysis in such scenarios within the Methods section and recommend that future studies, when obtaining additional behavioral and policy variable data, should further evaluate the incremental contribution of these variables to the model’s explanatory power. One probable potential mechanism is that persistent behavioral changes, such as widespread teleworking and sustained vigilance toward respiratory symptoms, may have weakened the pathways through which other environmental factors influence transmission by altering crowd gathering and contact patterns. This could have amplified the virus survival and transmission mechanisms associated with absolute temperature (Gao et al., 2025). Given the potential viral evolution and herd immunity dynamics, these conclusions warrant caution and require further validation through subtype-stratified data, genetic sequencing analysis, or serological evidence.

Another key finding of this study is the temporal heterogeneity in the influence of the environmental factors. During the COVID-19 pandemic, when NPI intensity was extremely high, the explanatory power of all environmental factors on influenza transmission was significantly weakened (Hussain et al., 2024; Zhang et al., 2025), with SHAP values clustered near zero. This phenomenon strongly suggests that when social contact networks are artificially suppressed to extremely low levels, the influence of natural drivers like meteorological conditions becomes “masked.” Thus, the intensity of social contact constitutes a fundamental prerequisite for transmission (Guo et al., 2015), while environmental factors act as modulators, functioning as either ‘accelerators’ or “decelerators”. In the post-pandemic phase, environmental influences re-emerged, although their distribution patterns differed from pre-pandemic levels (Zhang et al., 2025). In addition to the enhanced dominance of temperature, atmospheric pressure exhibited a more robust influence than pre-pandemic levels. This suggests that within the “new normal” characterized by shifts in population immunity and social behavior patterns, previously obscured transmission mechanisms linked to atmospheric circulation or indoor-outdoor pressure differentials may exist, warranting further investigation. This finding has significant implications for public health practices. Traditional influenza early warning models based on historical meteorological data may exhibit diminished performance in the post-pandemic era. Models must incorporate dynamic parameters that reflect the recovery of social contact levels and recalibrate the weights assigned to environmental variables.

This study had several key limitations that should be carefully considered when interpreting the results. First, based on observational surveillance and environmental data, it does not incorporate dynamic information on individual behavior, viral genomic variations, or population immunity levels. Therefore, the identified associations should not be directly interpreted as causal relationships (Yang et al., 2023). Second, the lack of sufficient subtype breakdown, genomic sequences, and serological data prevents quantifying the specific contributions of antigenic drift or “immunity debt” during the pandemic to the 2023–2024 influenza season pattern in the country. The overall lw explanatory power (R²) of the post-pandemic model suggests that unobserved non-meteorological factors, such as NPIs, population behavior changes, testing strategies, vaccine coverage, and viral evolution may significantly influence influenza variability. Therefore, the “contribution rates” of meteorological variables reported in this study based on the SHAP analysis should be understood as indicators of relative importance within the model, rather than measures of causal effects or absolute contributions. Third, this study was conducted in temperate continental climate zones, and its conclusions may have limited applicability to other climatic regions in the world. To more clearly distinguish the relative roles of meteorological drivers, virological factors, and immune history, future research should establish a multi-source data fusion analytical framework. This should integrate subtype-resolved case data, genetic sequences, antigen/genetic distance information, serological surveillance, vaccination coverage, and mobile communication or behavioral and NPIs indicators. This would enhance model interpretability and, through sensitivity analysis and counterfactual simulations, more precisely reveal influenza transmission dynamics and evolutionary trends within the complex, post-pandemic ecosystem.

## 5 Conclusion

In summary, this study provides preliminary observations that the COVID-19 pandemic in Lanzhou, China, not only disrupted influenza transmission in the short term but also reshaped its long-term epidemiology. Influenza has gradually transitioned from a complex pattern traditionally co-regulated by multiple environmental factors and accompanied by subtype alternation to a new phase primarily influenced by temperature, with simplified overall environmental drivers. This shift underscores the dynamic evolution of infectious disease ecology. Future surveillance and control strategies must be more flexible and forward-looking, continuously monitoring changes in the interactive network among social behaviors, environmental conditions, viral evolution, and host immunity, to respond promptly to potential future epidemiological shifts.

## Data Availability

The original contributions presented in the study are included in the article/**Supplementary Material**. Further inquiries can be directed to the corresponding authors.
