# Supplementary material for: A study on the climate-driven spatiotemporal dynamics of influenza in Lanzhou spanning the COVID-19 era
Source: Front Cell Infect Microbiol. 2026 Feb 23;16:1765305. doi: 10.3389/fcimb.2026.1765305 (PMC12968309; doi:10.3389/fcimb.2026.1765305)
Supplement: Supplementary file 1 [file Table1.docx]

Table S1 Evaluation Metrics for XG-Boost Model Training

| Period | *R²* | *RMSE* |
| --- | --- | --- |
| Pre-COVID | 0.4841 | 0.1350 |
| COVID | 0.1525 | 0.1600 |
| Post-COVID | 0.1760 | 0.1354 |

Table S2 Differential Analysis of Positive Rates of Influenza Across Distinct Periods

| Period | *P*-Value |
| --- | --- |
| Pre- vs COVID | ＜0.05 |
| Pre- vs Post- | 1.000 |
| COVID vs Post- | ＜0.05 |

*Noted: Three-group comparisons performed using Kruskal-Wallis test or ANOVA; pairwise comparisons using the Wilcoxon signed-rank test, and correct for multiple comparisons using the Bonferroni method., with statistical significance set at P<0.05.*

Table S3 Differential Analysis of Positive Rates of Influenza Across Distinct Seasons

| Season | *X^2^* | *P*-Value |
| --- | --- | --- |
| Spring | 0.762 | 0.6830 |
| Summer | 1.077 | 0.5837 |
| Autumn | 17.995 | ＜0.001 |
| Winter | 17.865 | ＜0.001 |

Table S4 Contribution (%) of Environmental Factors across Different Periods for Influenza

| Environmental factors | Pre-COVID | | COVID-19 | Post-COVID |
| --- | --- | --- | --- | --- |
| Temperature | 38.40 | 39.05 | | 47.55 |
| Atmospheric Pressure | 12.89 | 26.07 | | 12.46 |
| Relative Humidity | 20.93 | 8.75 | | 9.29 |
| Sunshine Duration | 5.99 | 10.02 | | 7.25 |
| Precipitation | 10.06 | 4.59 | | 7.39 |
| Temperature Range | 6.92 | 8.54 | | 8.39 |
| Wind Speed | 4.80 | 2.99 | | 7.68 |
